# Remarkable Gloves

**Published:** 1875-11

**Authors:** 


### REMARKABLE GLOVES.

We spent a week or two at Littleton, N. H., last summer, on our White Mountain trip, and made the acquaintance of the Messrs. Parker, who are the leading men of the Littleton Saranac Buck-Glove Company. They buy buckskins from all sources, tan them by a peculiar process, and convert them into gloves for men and women. We bought a few pairs of the gloves, and are now wearing them. They are very comfortable, wonderfully strong, and, what seems remarkable, quite waterproof,— qualities which make them just the thing for driving. The tanning process used is said to strengthen rather than weaken the skin. It struck us that buckskin so treated would make very comfortable and durable boots and shoes; we have therefore requested the Messrs. Parker to select a good hide, color it black instead of leaving it of the natural tan-color, and send it to us. If we ever receive it, we shall have it made up into foot-clothing by Joel McComber, and will tell our readers what we think of the new product. Made into gloves it is capital, being more cloth-like upon the hand than any glove we have ever worn.

Labor.—It is to labor, and labor only, that man owes everything possessed of exchangeable value. Labor is the talisman that has raised him from the condition of the savage; that has changed the desert and the forest into cultivated fields; that has covered the earth with cities, and ocean with ships; that has given us plenty, comfort, and elegance, instead of want, misery, and barbarism.

